# Supplementary material for: Three Alkaloids from an Apocynaceae Species, Aspidosperma spruceanum as Antileishmaniasis Agents by In Silico Demo-case Studies
Source: Plants (Basel). 2020 Aug 3;9(8):983. doi: 10.3390/plants9080983 (PMC7465237; doi:10.3390/plants9080983)
Supplement: Supplementary file 1 [file plants-09-00983-s001.pdf]

SUPPEMENTARY MATERIAL

Table S1. Metabolites from *Aspidosperma spruceanum*.

Metabolites

a. (+)-Aspidocarpina.
b. Desacetilaspidocarpina.
c. N-acetil-16,17-dihidroxiaspidospermidina (Des-O-metilaspidocarpina).
d. N-propionil-16,17-dihidroxiaspidospermidina.

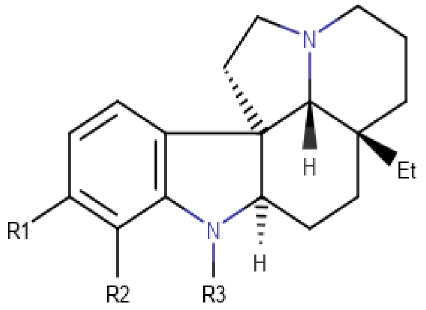

a: R1 =OMe; R2 =OH; R3 =COMe
b: R1 =OMe; R2 =R3 =H
c: R1 =R2 =OH; R3 =COMe
d: R1 =R2 =OH; R3 =COEt

e. Aspidoalbina
f. O-metilaspidoalbina
g. N-acetil-N-despropionilaspidoalbina
h. Aspidolimidina
i. Des-O-metilaspidolimidina
j. Fendlerina

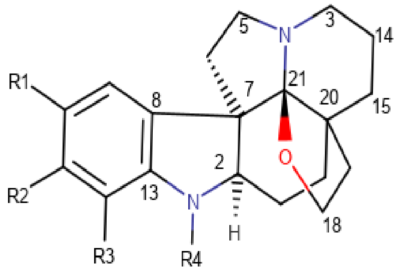

e: R1 =R2 =OMe; R3 =OH; R4 =COEt
f: R1 =R2 =R3 =OMe; R4 =COEt
g: R1 =R2 =OMe; R3 =OH; R4 =COMe
h: R1 =H; R2 =OMe; R3 =OH; R4 =COMe
i: R1 =H; R2 =R3 =OH; R4 =COMe
j: R1 =H; R2 =OMe: R3 =OH; R4 =COEt

k. 15-metoxilimaspermina
l. N-acetil-limaspermina (limapodina)

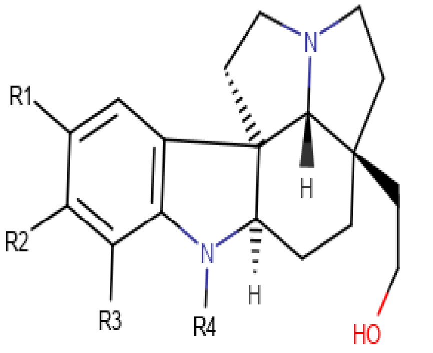

k: R1 =OMe; R2 =H; R3 =OH; R4= COEt
l: R1 =R2 =H; R3 =OH; R4 =COMe

m. (+)- Quebrachamina
n. (-) Quebrachamina

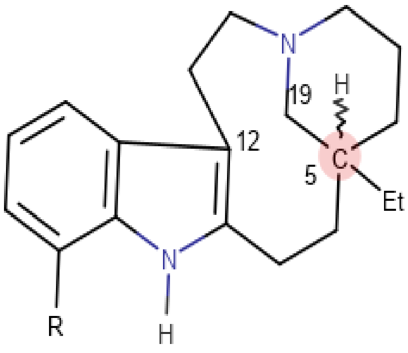

m: H-5α
n: H-5β

o. 16(S)-Isositsirikina

p. 16(R)-epi-Isositsirikina

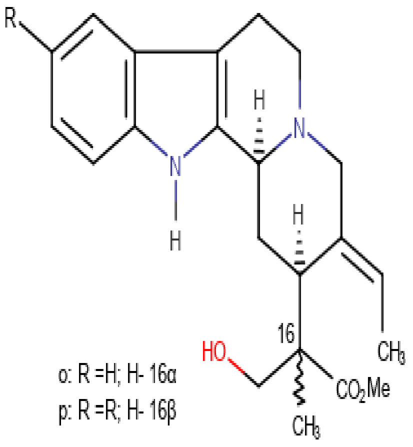

(+)-Aspidospermina

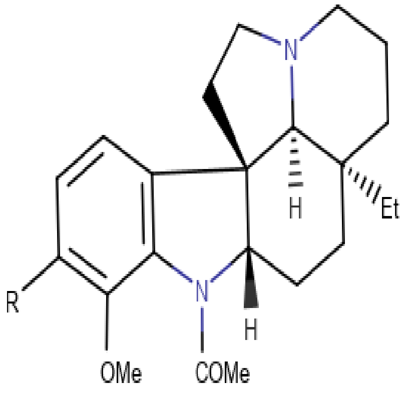

R= H

Tubotaiwina (dihidrocondilocarpina)

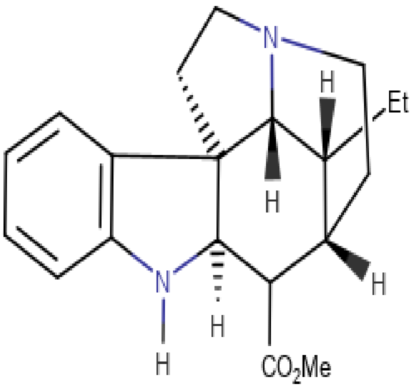

21-Oxoaspidoalbina

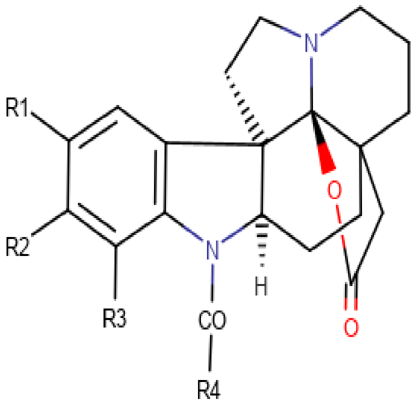

R1 =R2 =OMe; R3 =OH; R4 =Me

DHFR-TS (PDB ID 3INV) and TS (PDB ID 1HW3)

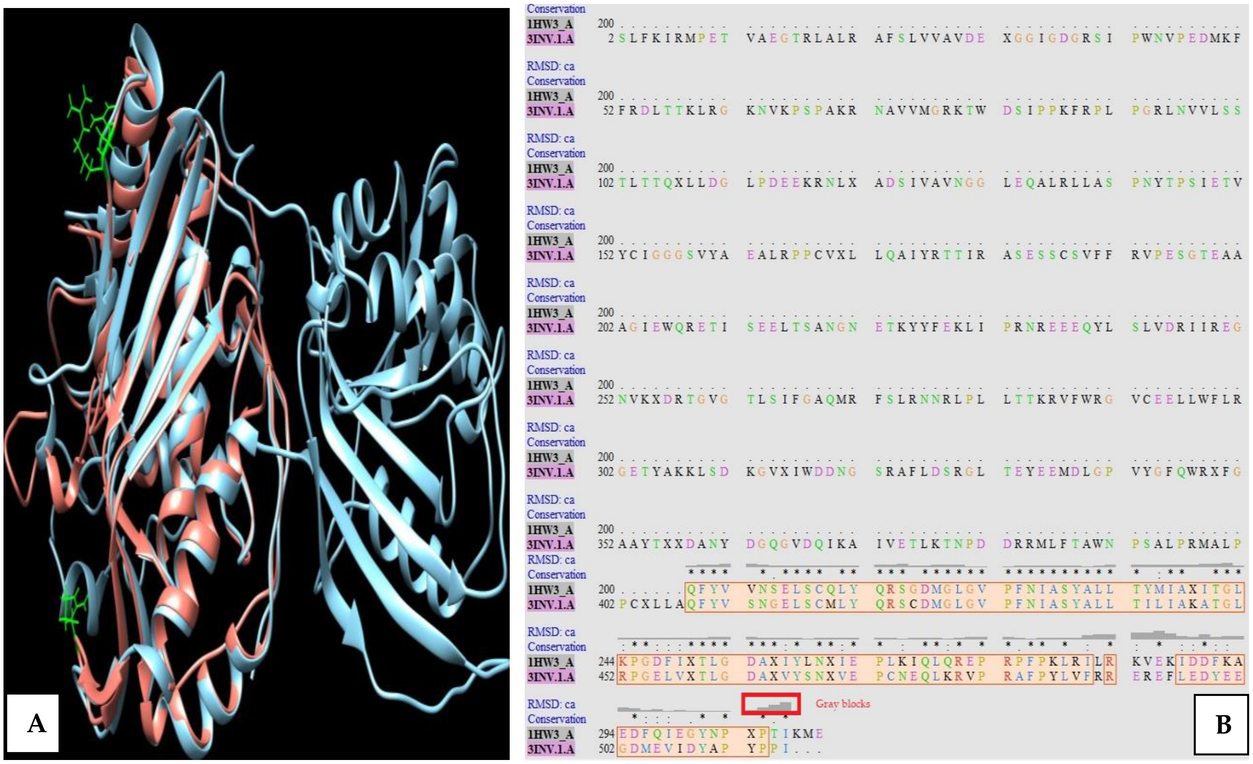

Figure S1. Structural Models of DHFR-TS (3INV) and TS (1HW3) targets. (**A**) Overlapping of Leishmania (sky blue) and *H. sapiens* (salmon) enzymes. Green zones indicate position of interest in humans. (**B**) Alignment sequence. The aligned regions are indicated in the colored boxes, root mean square deviation (RMSD) as gray blocks, "*" identical or preserved residues; ":" Preserved substitutions and "." Semi-preserved substitutions.

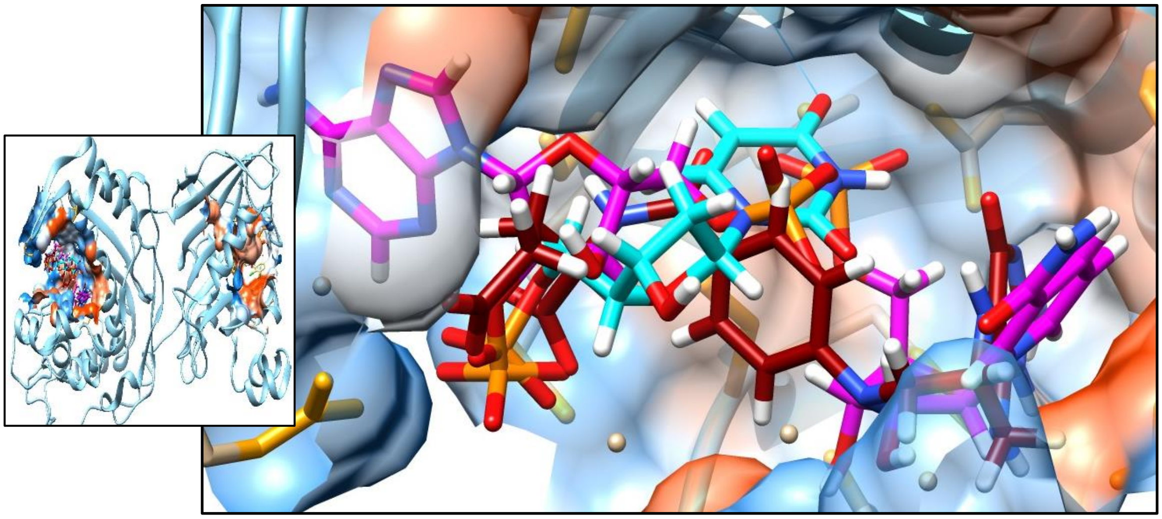

Figure S2. Complex of DHFR-TS (PDB ID: 3INV) with three physiological ligands: NADP (magenta), dUMP (cyan) and THF (dark red). Small box, total vision, and big box, augmented vision of the protein surface in transparency style of USCF Chimera program.

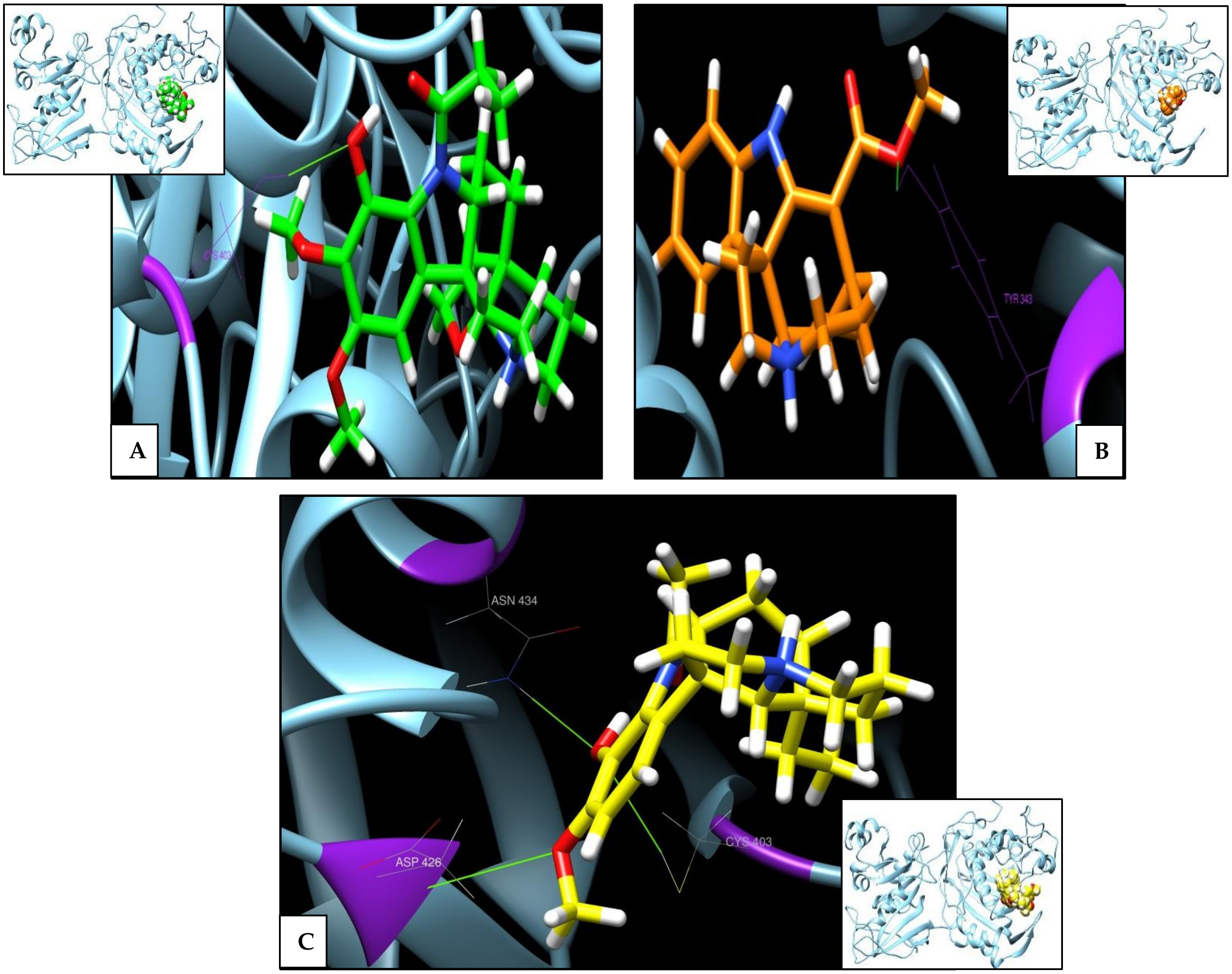

Figure S3. 3D molecular interactions of the complexes of DHFR-TS (PDB ID 3INV) with (**A**) APA in Cys403. (**B**) TBT in Tyr343 and (**C**) APC in the residues Cys403, Asp426 and Asn434. Small box, total vision, and big box, augmented vision, where the protein is represented in cartoon shapes (secondary structures) of sky blue, with H bonds in green and ligands in the form of colored sticks per element.

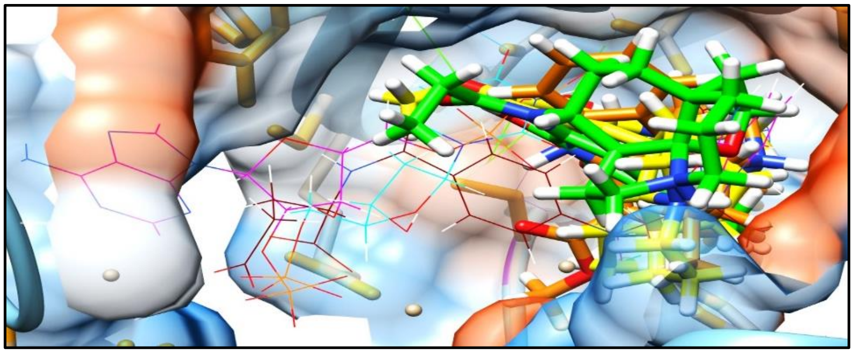

Figure S4. 3D molecular interaction of the complex DHFR-TS (3INV) with dUMP, THF and NADP represented by wire representations; APC (**yellow**), APA (**green)** and TBT (**orange**) by stick representations;

and the protein surface represented by blue for the more hydrophilic, and **white** (neutral), **orange** and **red** for the more lipophilic regions.

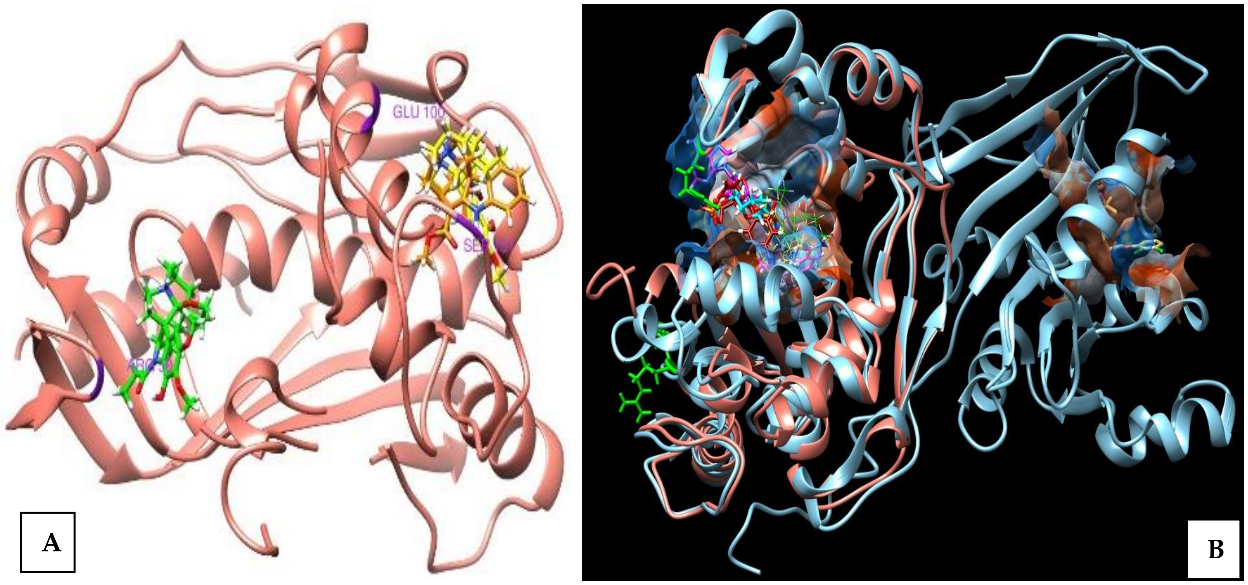

Figure S5. 3D molecular structure of human TS and Leishmania DHFR-TS. (**A**) Position of metabolites APC (**yellow**), APA in Arg50 (**green**) and TBT in Ser151, Glu100 (**orange**) in TS human target. (**B**) Superposition of active sites and ligands of the enzymes DHFR-TS (**sky blue**) and TS (**salmon**) made with UCSF Chimera Software.

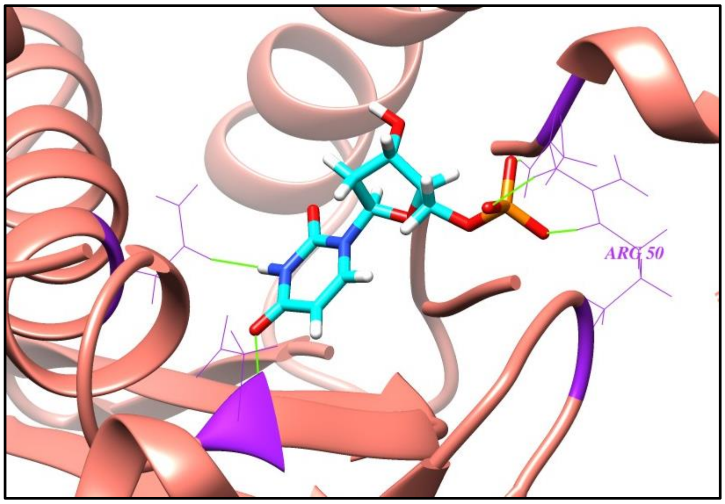

Figure S6. Docking molecular structure between TS human target (PDB ID 1HW3) y dUMP (**cyan**). The residues involved are marked with purple color and the binding energy is of -10.44 kcal/mol.

PK (PDB ID 3HQN) y PKLR (PDB ID 2VGB)

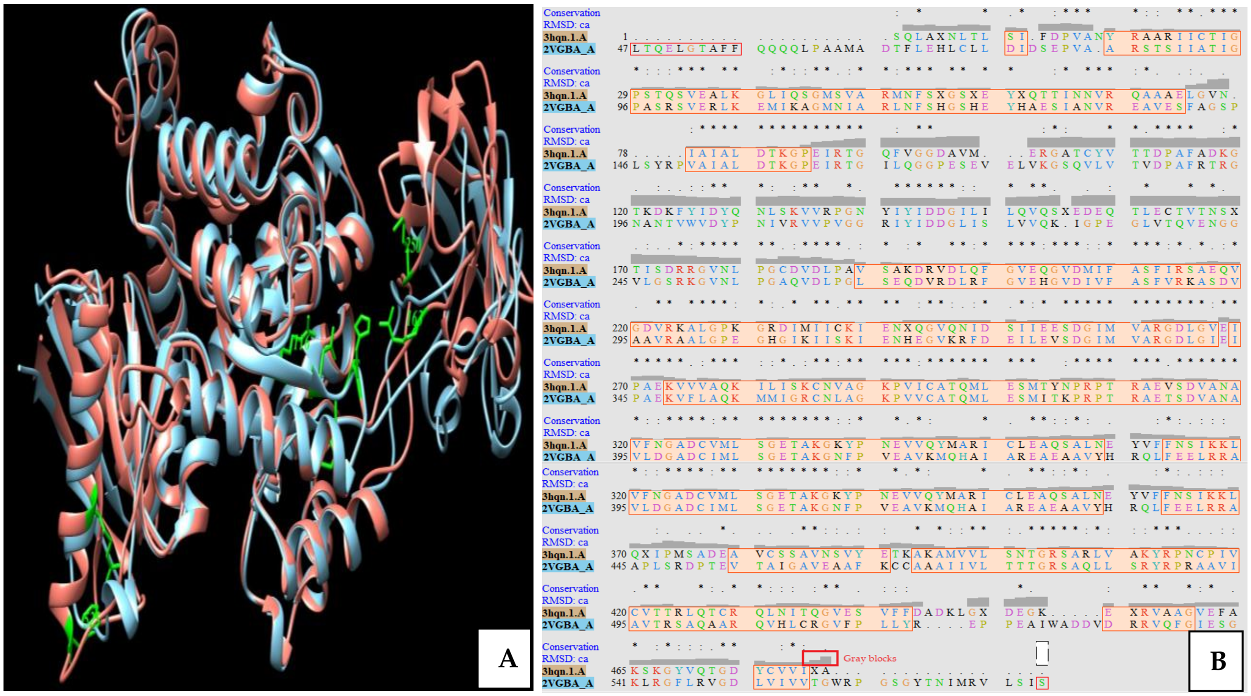

Figure S7. Structural Models of PTR1 (1E7W) and DHR (3O4R) targets. (**A**) Overlapping of Leishmania (sky blue) and *H. sapiens* (salmon) enzymes. Green zones indicate position of interest in humans. (**B**) Alignment sequence. The aligned regions are indicated in the colored boxes, root mean square deviation (RMSD) as gray blocks, "*" identical or preserved residues; ":" Preserved substitutions and "." Semi-preserved substitutions.

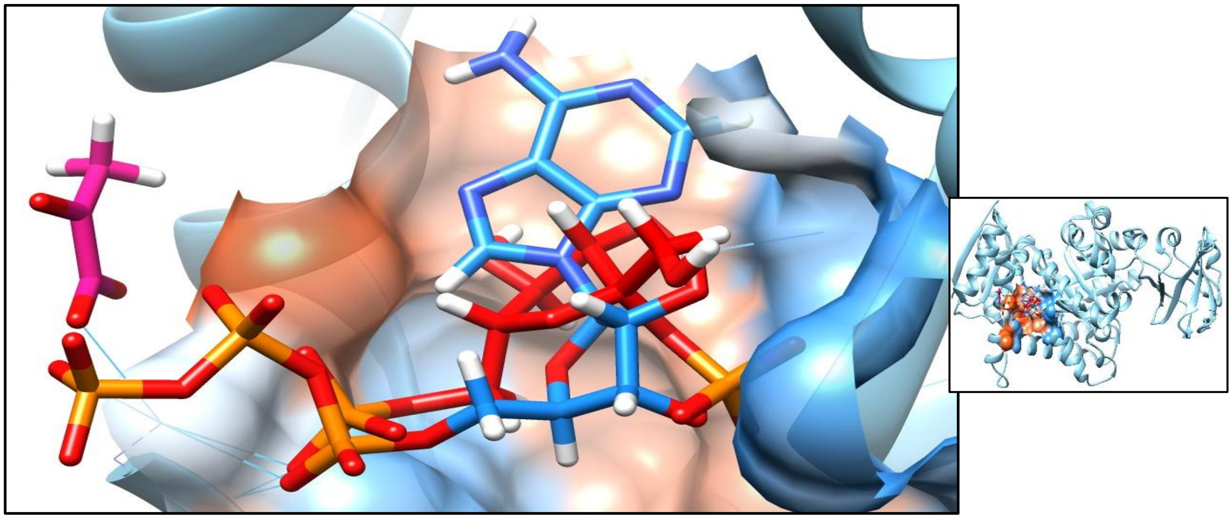

Figure S8. Complex of PK (PDB ID: 3HQN) with three physiological ligands: PVT (**deep pink**), FDP (**red**) and ATP (**dodger blue**). Small box, total vision, and big box, augmented vision of the protein surface in transparency style of USCF Chimera program.

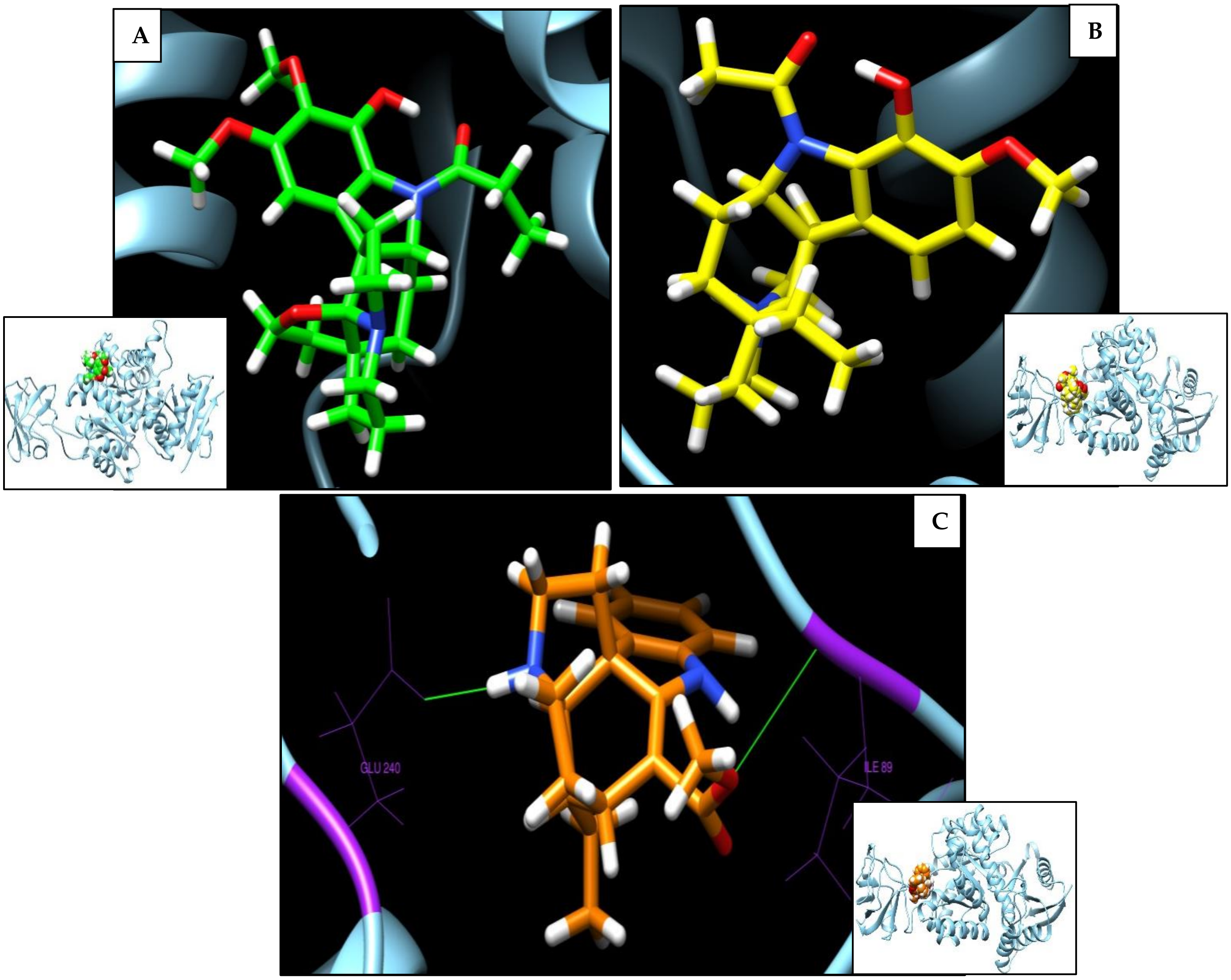

Figure S9. 3D molecular interactions of the complexes of PK (PDB ID 3HQN) with (**A**) APA, (**B**) APC and (**C**) TBT in Glu240 and Ile89. Small box, total vision, and big box, augmented vision, where the protein is represented in cartoon shapes (secondary structures) of sky blue, with H bonds in green and ligands in the form of colored sticks per element.

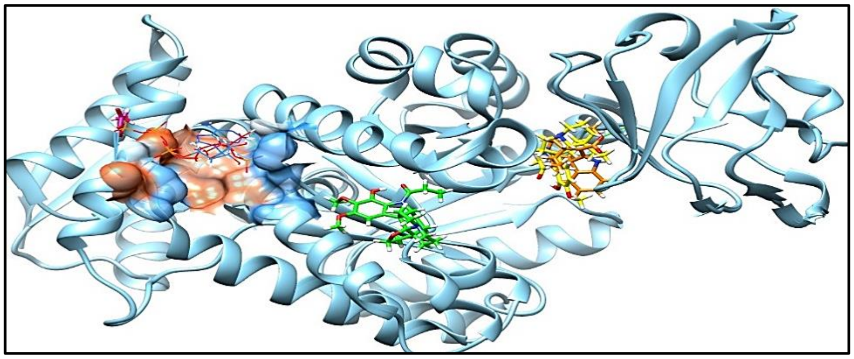

Figure S10. 3D molecular interaction of the complex PK (3HQN) with PVT, FDP and ATP represented by wire representations; APC (**yellow**), APA (**green)** and TBT (**orange**) by stick representations; and the

protein surface represented by blue for the more hydrophilic, and **white** (neutral), **orange** and **red** for the more lipophilic regions.

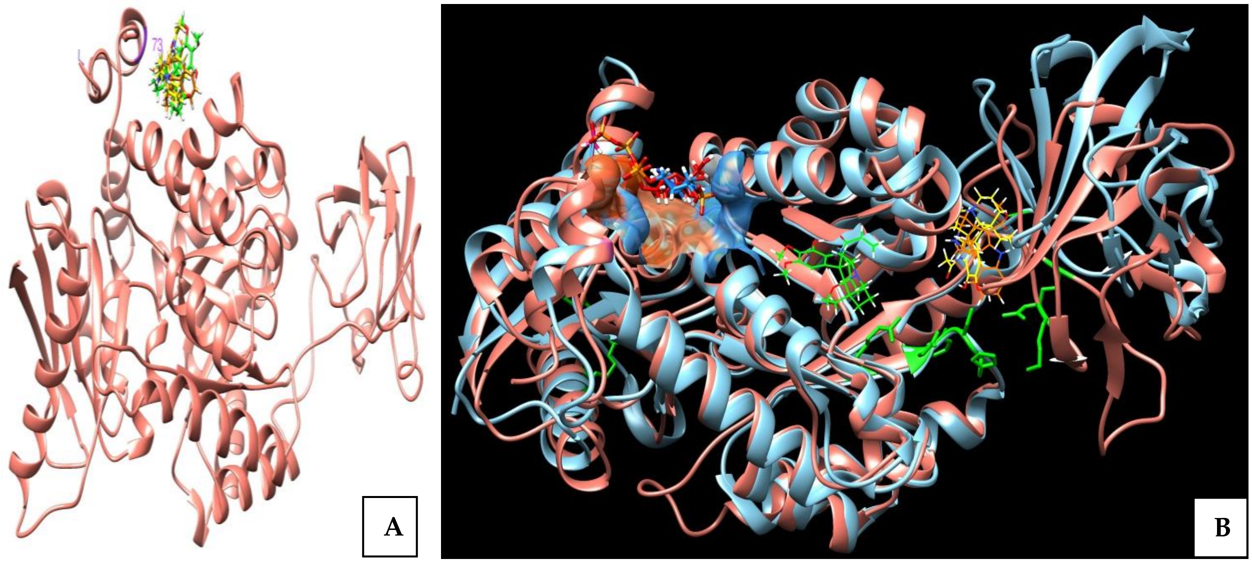

Figure S11. 3D molecular structure of human PK and Leishmania PKLR. (**A**) Position of metabolites APC (**yellow**), APA (**green**) and TBT (**orange**) in PK human target. (**B**) Superposition of active sites and ligands of the enzymes PK (**sky blue**) and PKLR (**salmon**) made with UCSF Chimera Software.

HGPRT L. (PDB ID 1PZM) y HGPRT (PDB ID 1BZY)

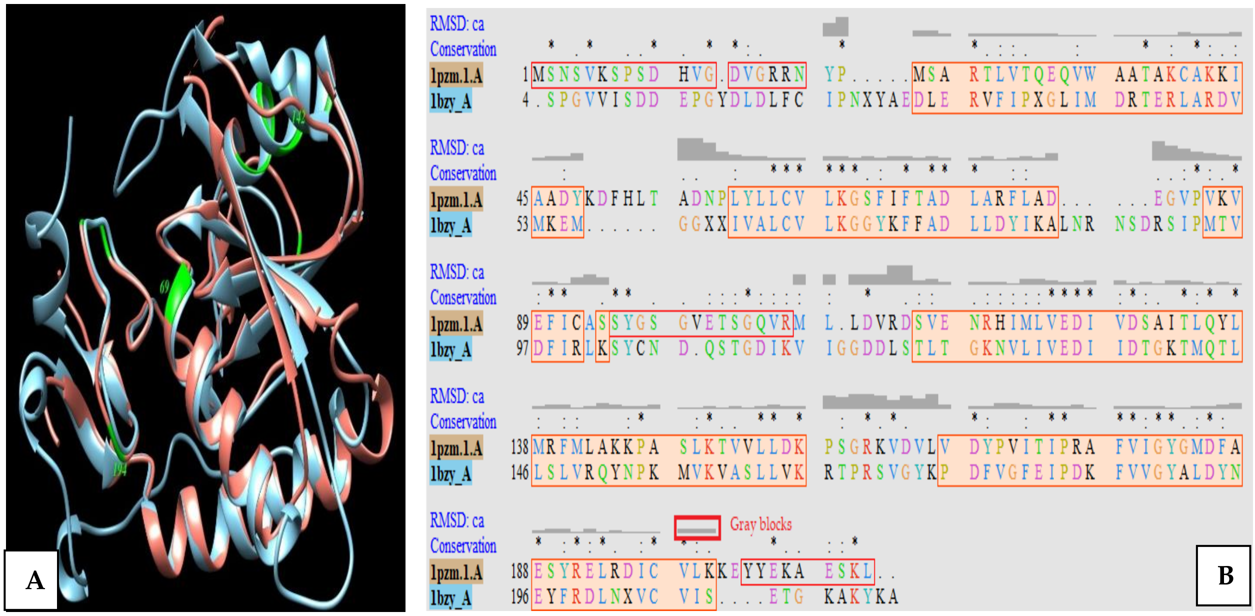

Figure S12. Structural Models of HGPRT (1PZM) and HGPRT (1BZY) targets. (**A**) Overlapping of Leishmania (sky blue) and *H. sapiens* (salmon) enzymes. Green zones indicate position of interest in humans. (**B**) Alignment sequence. The aligned regions are indicated in the colored boxes, root mean square deviation (RMSD) as gray blocks, "*" identical or preserved residues; ":" Preserved substitutions and "." Semi-preserved substitutions.

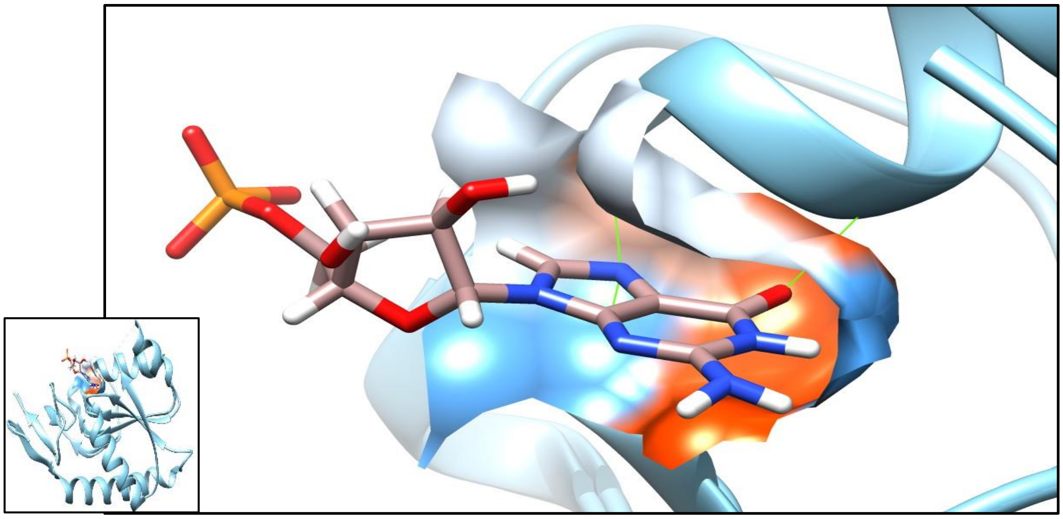

Figure S13. Complex of HGPRT (PDB ID: 1PZM) with 5GP. The residues involved are Arg176, Ala131, Asp 129, Thr 133, Leu 134 and Glu125. Small box, total vision, and big box, augmented vision of the protein surface in transparency style of USCF Chimera program.

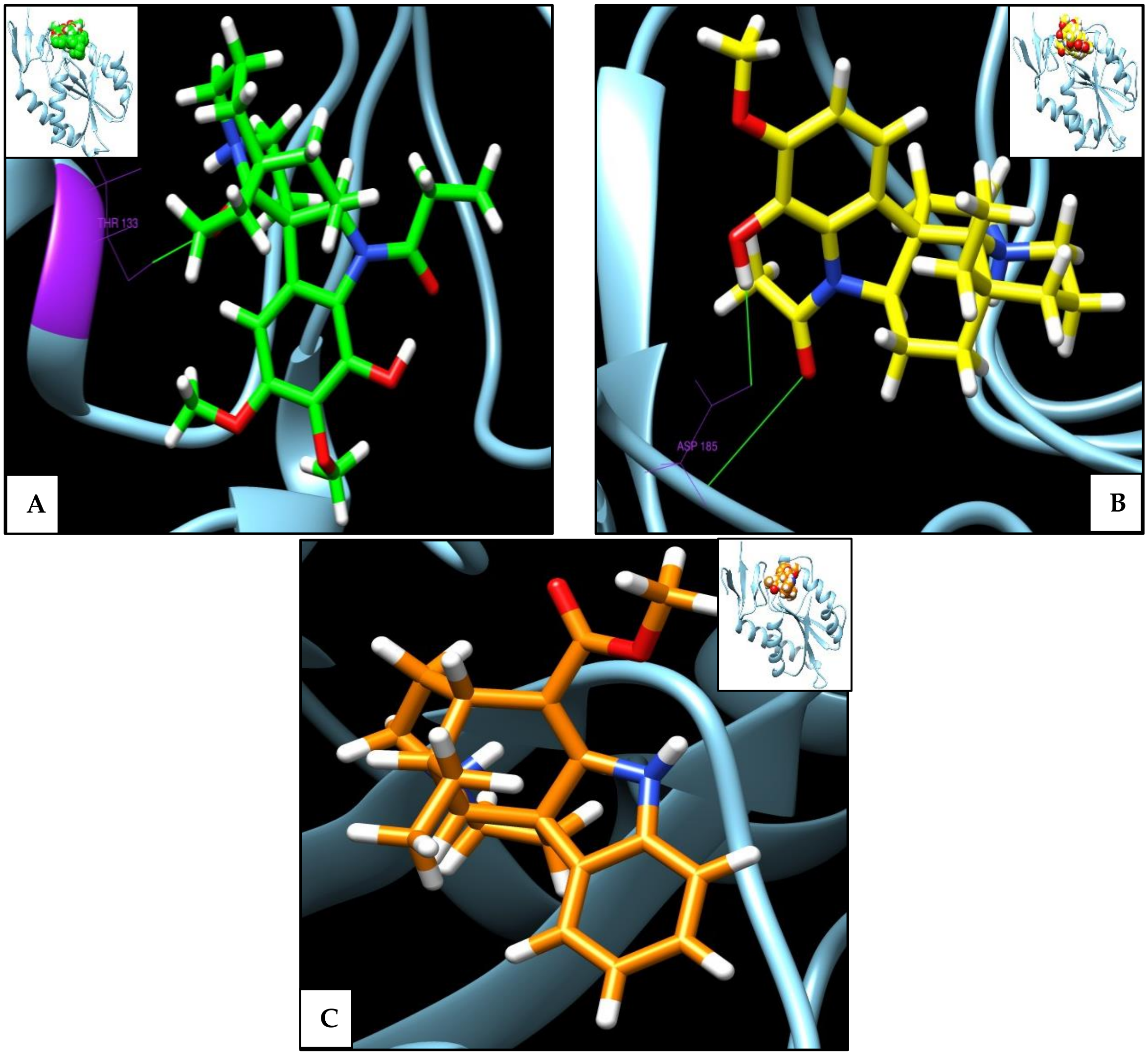

Figure S14. 3D molecular interactions of the complexes of HGPRT (PDB ID 1PZM) with **(A)** APA in Thr133, **(B)** APC in Asp185 and **(C)** TBT. Small box, total vision, and big box, augmented vision, where the protein is represented in cartoon shapes (secondary structures) of sky blue, with H bonds in green and ligands in the form of colored sticks per element.

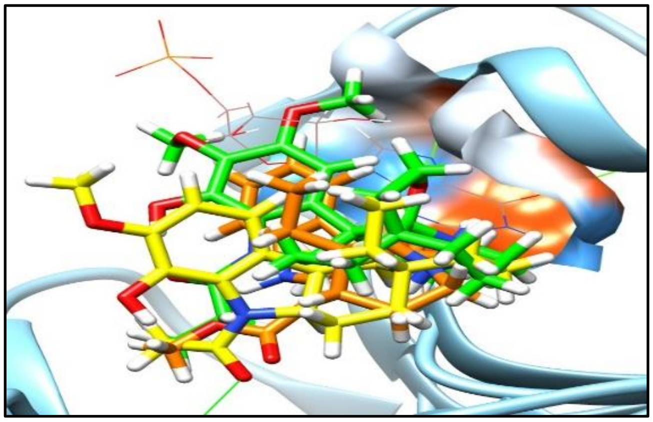

Figure S15. 3D molecular interaction of the complex HGPRT (1PZM) with 5GP represented by wire representations; APC (**yellow**), APA (**green)** and TBT (**orange**) by stick representations; and the protein surface represented by blue for the more hydrophilic, and **white** (neutral), **orange** and **red** for the more lipophilic regions.

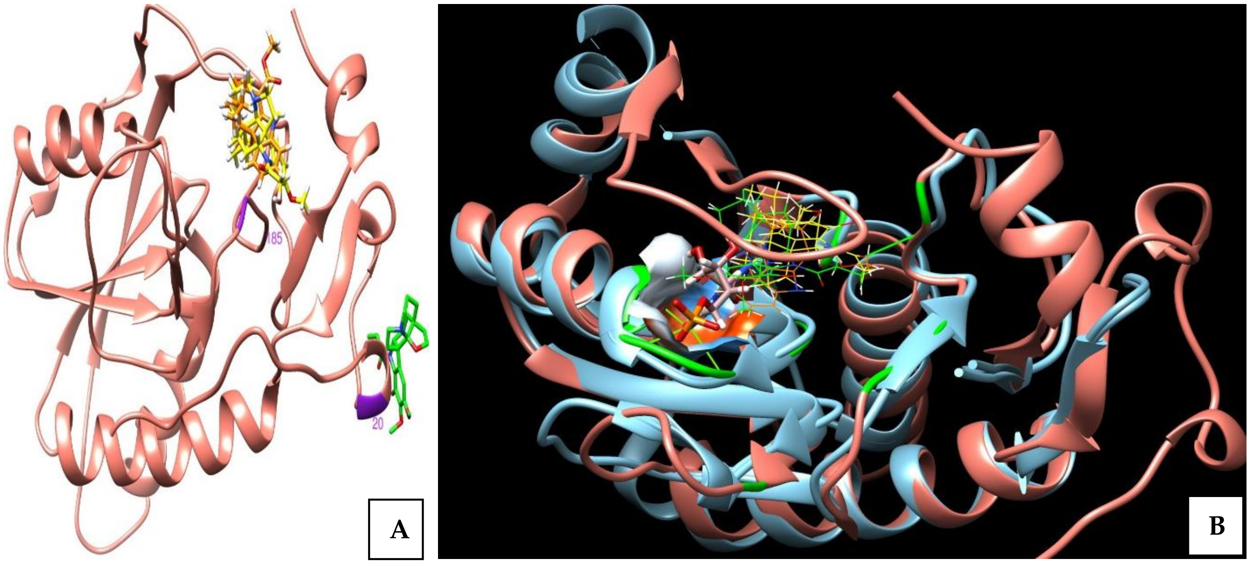

Figure S16. 3D molecular structure of human and Leishmania HGPRT. (**a**) Position of metabolites APC (yellow), APA (green) and TBT (orange) in HGPRT human target. (**b**) Superposition of active sites and ligands of the enzymes HGPRT (sky blue) and HGPRT (salmon) made with UCSF Chimera Software.

SQS L. (PDB ID 3WCA) y SQS (PDB ID 1EZF)

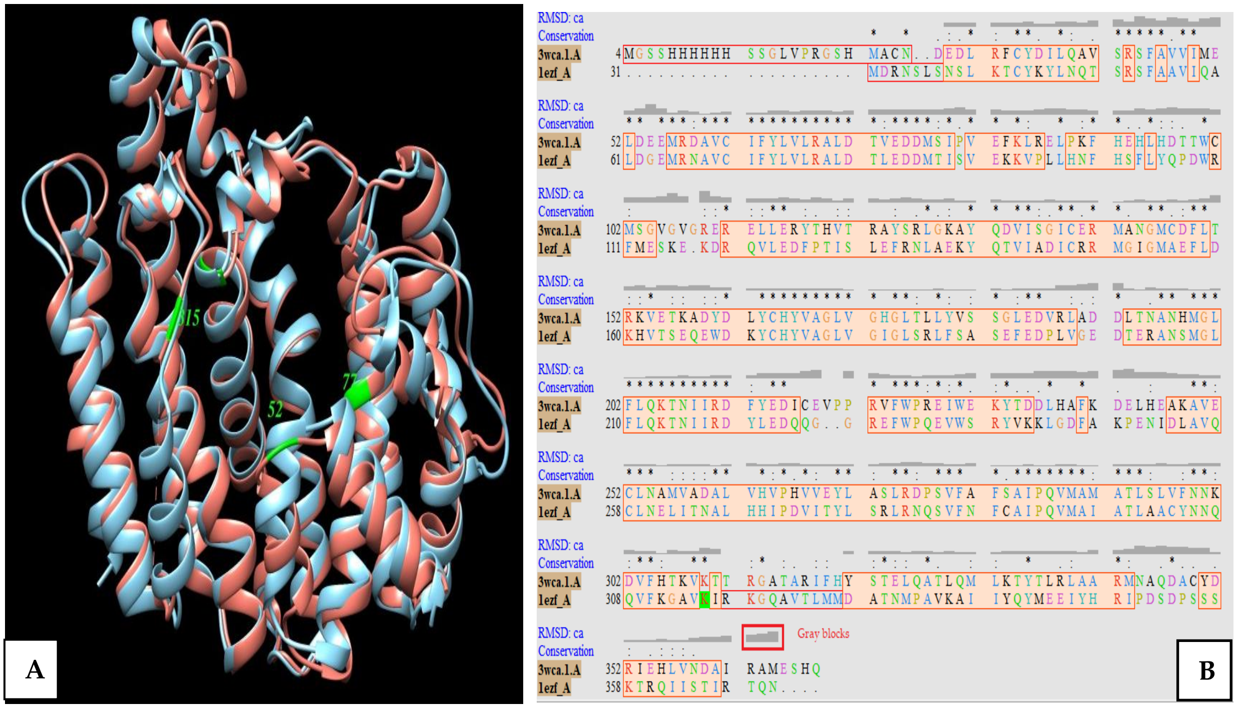

Figure S17. Structural Models of SQS leishmania (3WCA) and SQS human (1EZF) targets. (**A**) Overlapping of Leishmania (sky blue) and *H. sapiens* (salmon) enzymes. Green zones indicate position of interest in humans. (**B**) Alignment sequence. The aligned regions are indicated in the colored boxes, root mean square deviation (RMSD) as gray blocks, "*" identical or preserved residues; ":" Preserved substitutions and "." Semi-preserved substitutions.

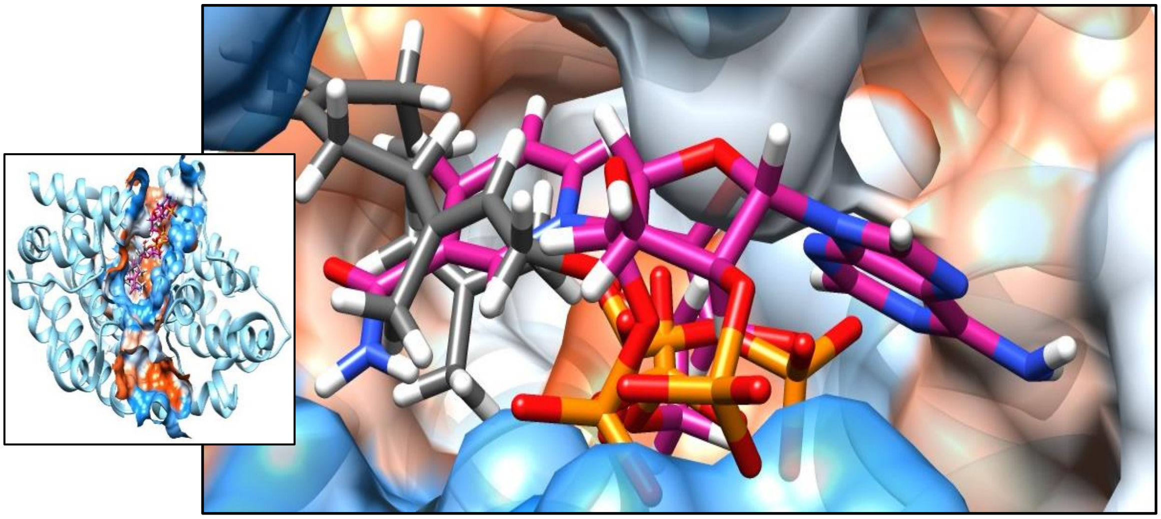

Figure S18. Complex of SQS (PDB ID: 3WCA) with two physiological ligands: NADPH (**violet red**) and FSP (**dim gray**). Small box, total vision, and big box, augmented vision of the protein surface in transparency style of USCF Chimera program.

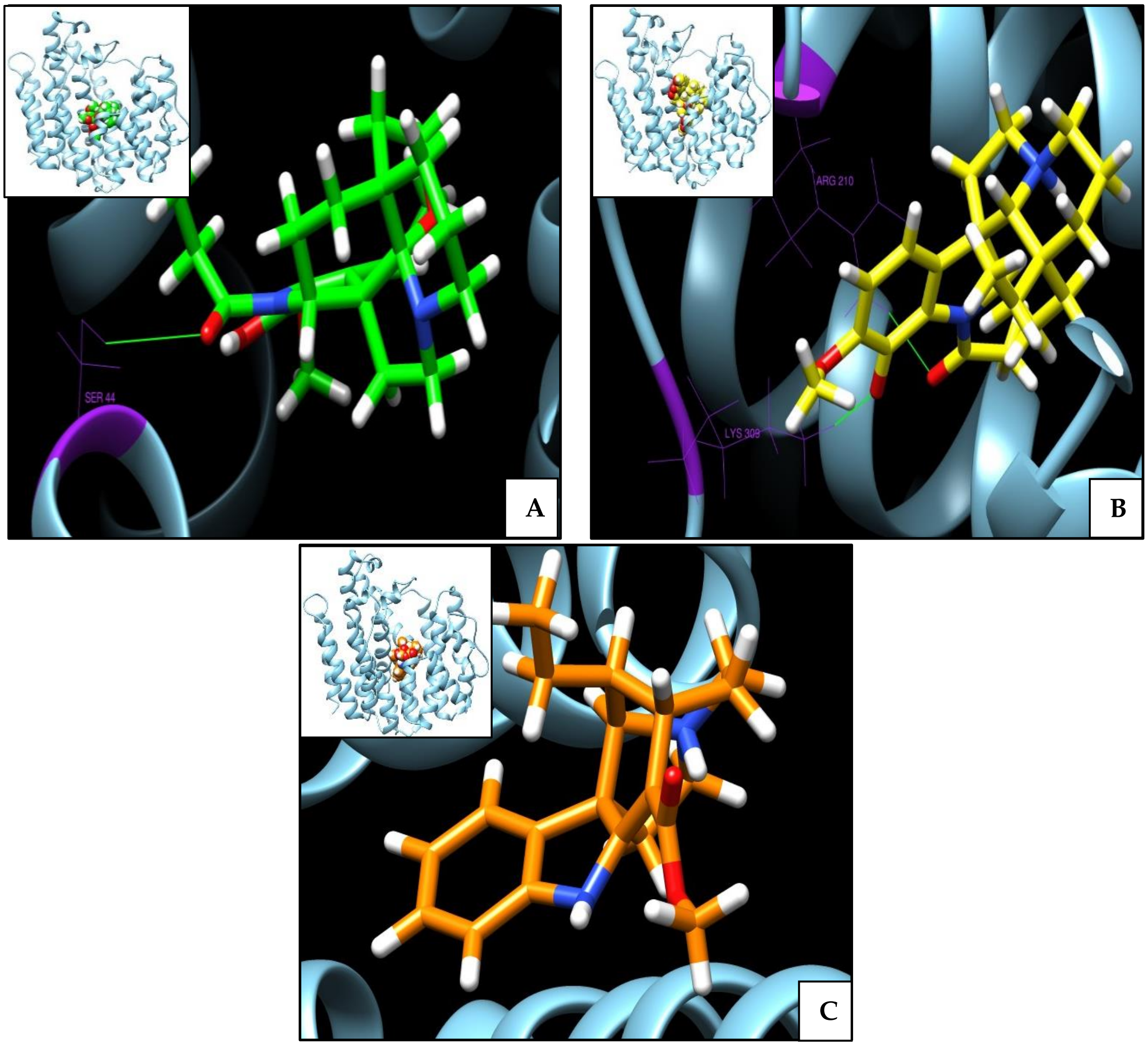

Figure S19. 3D molecular interactions of the complexes of SQS (PDB ID 3WCA) with **(A)** APA in Ser44, **(B)** APC in Lys309, Arg210; and **(C)** TBT. Small box, total vision, and big box, augmented vision, where the protein is represented in cartoon shapes (secondary structures) of sky blue, with H bonds in green and ligands in the form of colored sticks per element.

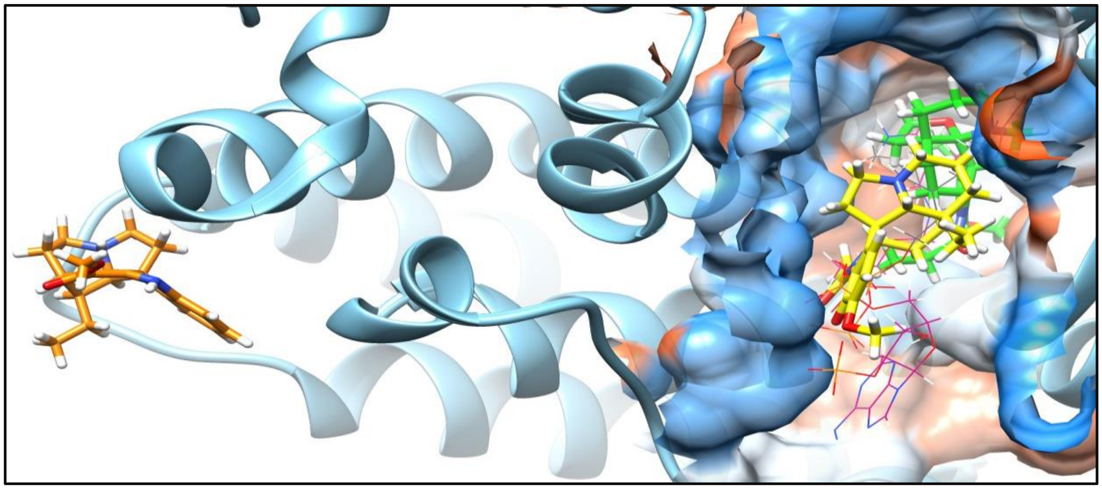

Figure S20. 3D molecular interaction of the complex SQS (3WCA) with FPS and NADPH, represented by wire representations; APC (**yellow**), APA (**green)** and TBT (**orange**) by stick representations; and the protein surface represented by blue for the more hydrophilic, and **white** (neutral), **orange** and **red** for the more lipophilic regions.

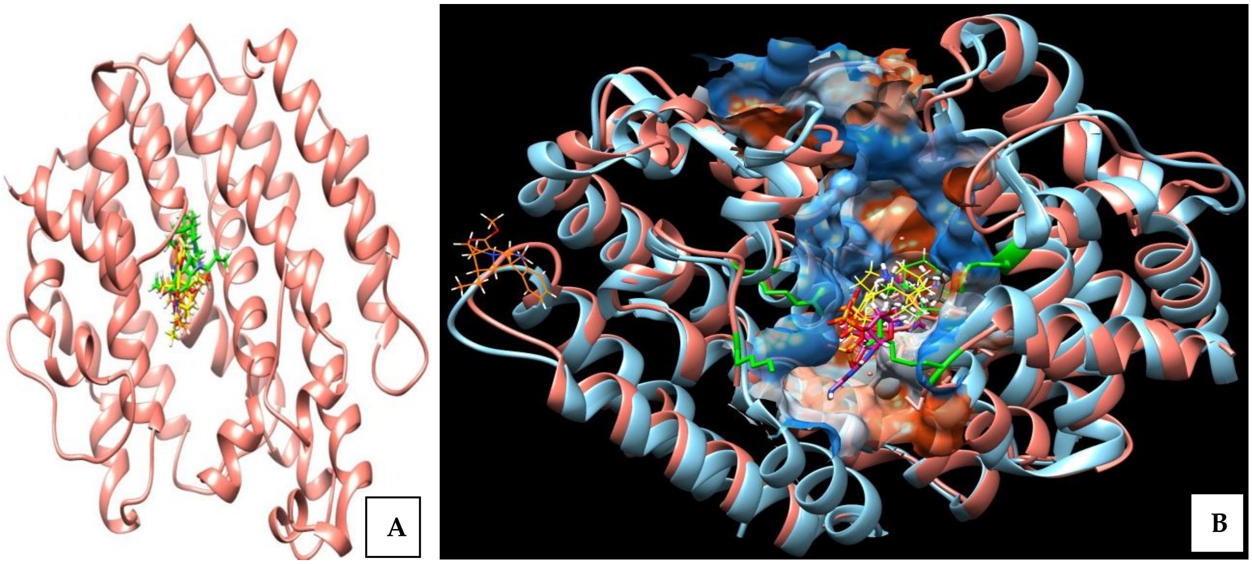

Figure S21. 3D molecular structure of human and Leishmania SQS. (**a**) Position of metabolites APC (yellow), APA (green) and TBT (orange) in SQS human target. (**b**) Superposition of active sites and ligands of the enzymes SQS (sky blue) and SQS (salmon) made with UCSF Chimera Software.
